# Magnetic resonance imaging in the second trimester as a complement to ultrasound for diagnosis of fetal anomalies

**DOI:** 10.1177/20584601241248820

**Published:** 2024-05-11

**Authors:** Frida Cederlund, Ove Axelsson, Sara Desmond, Hashem Amini, Johan Wikström

**Affiliations:** 1 Centre for Clinical Research Sörmland, 8097 Uppsala University, Eskilstuna, Sweden; 2 Department of Women’s and Children’s Health, 8097 Uppsala University, Uppsala, Sweden; 3 Department of Surgical Sciences, Neuroradiology, 8097 Uppsala University, Uppsala, Sweden

**Keywords:** Fetal diagnosis, fetal magnetic resonance imaging, ultrasound, second trimester, fetal anomalies, central nervous system anomalies

## Abstract

**Background:**

Fetal ultrasound has limitations, especially if the patient is obese or in cases with oligohydramnios. Magnetic resonance imaging (MRI) can then be used as a complement, but only few studies have focused on examinations in the second trimester.

**Purpose:**

To validate MRI as a complement to diagnose fetal anomalies in the second trimester.

**Material and Methods:**

This retrospective study retrieved data from January 2008 to July 2012 from the Fetal Medicine Unit and Department of Radiology at Uppsala University Hospital. Ultrasound and MRI findings were reviewed in 121 fetuses in relation to the final diagnosis, including postpartum follow-up and autopsy results.

**Results:**

Of the 121 fetuses, 51 (42%) had a CNS anomaly and 70 (58%) a non-CNS anomaly diagnosed or suspected. MRI provided additional information in 21% of all cases without changing the management and revealed information that changed the management of the pregnancy in 13%. When a CNS anomaly was detected or suspected, the MRI provided additional information in 22% and changed the management in 10%. The corresponding figures for non-CNS cases were 21% and 16%, respectively. The proportion of cases with additional information that changed the management was especially high in patients with a BMI >30 kg/m^2^ (25%) and in patients with oligohydramnios (38%). In five cases in category III, false-positive ultrasound findings were identified.

**Conclusions:**

MRI in the second trimester complements ultrasound and improves diagnosis of fetal CNS- and non-CNS anomalies especially when oligohydramnios or maternal obesity is present.

## Introduction

A great majority of pregnant women in Sweden, today, has an ultrasound examination around 18 postmenstrual weeks. One purpose with this examination is to detect or rule out fetal anomalies.^1^ In Sweden, a patient can decide on termination of pregnancy up to 18 weeks + 0 days. Thereafter, the patient must apply to the Swedish National Board of Health and Welfare to be allowed to terminate her pregnancy. A majority of late terminations is performed due to severe or lethal fetal anomalies. When a fetal anomaly is suspected or detected, it is of utmost importance that the diagnosis is as accurate as possible to be able to give correct information on the prognosis for the fetus. Correct information is a prerequisite for the patient to make a well-grounded decision if she wants to continue the pregnancy or not.

Fetal ultrasound has limitations, especially if the patient is obese or in cases with oligohydramnios.^2^ Magnetic resonance imaging (MRI) can then be used as a complement to ultrasound.^3–6^ MRI is highly accepted by patients when a fetal anomaly is suspected^7^ and is not shown to have any harmful effects on the fetus when used without administration of contrast media.^8^ Few studies on fetal MRI have focused on examinations in the second trimester, which is of special interest in countries with a similar legislation concerning pregnancy terminations as Sweden. We have previously performed minor studies on unselected cases focusing on the contribution of MRI as a complement to ultrasound to improve diagnosis of fetal anomalies in the second trimester.^9,10^ These studies have provided us with new insights concerning the indications and usefulness for fetal MRI, but the findings have not been validated on other populations.

The aim of this study is to validate MRI as a complementary tool to diagnose fetal anomalies in the second trimester.

## Material and methods

This retrospective study has retrieved data from January 2008 to July 2012 from the Fetal Medicine Unit and Department of Radiology at Uppsala University Hospital. This period was chosen as it directly followed the time of our previous studies. Thus, the equipment and technique for the ultrasound and MRI examinations were almost unchanged but the indications for fetal MRI were affected by our previous results. The majority of the participants were pregnant women in the second trimester where the routine ultrasound identified or suspected a fetal anomaly. Two patients participated due to previous pregnancies with fetal anomalies (corpus callosum agenesis and Joubert’s syndrome) with risk of recurrence. All patients were informed about the study and gave their consent to participate, both verbally and in writing.

In total, 146 patients were recruited fromUppsala county or as referrals from other hospitals in Sweden. Twenty-six patients were excluded, 23 because the MRI examination was performed in the third trimester. One patient underwent MRI before the ultrasound and two patients were excluded due to lack of information in their records. Thus, the study included 120 patients but 121 fetuses. Nine twin pregnancies were included and in one of these both fetuses had suspected anomalies.

The ultrasound was performed by five obstetricians specialized in fetal medicine, meaning at least 5 years experience of obstetric ultrasound, using commercially available real-time machines as previously described.^10^ The MRI examinations were performed on a 1.5 T Scanner (Gyroscan ACS Intera, Philips Medical Systems, Best, the Netherlands) using a pelvic phased array coil. T2-weighted images were acquired in the three main planes of the fetus (sagittal, coronal, and axial) using a Single-Shot Turbo Spin Echo (SS-TSE) sequence with a slice thickness of 3 mm. In addition, a T1-weighted sequence was performed in the axial plane with a slice thickness of 5 mm. Imaging was performed during free breathing with respiratory gating to avoid artefacts. In addition, some patients were also examined with 2D- or 3D-steady-state free precession (SSFP) sequences (Balanced-fast field echo [FFE], Philips Medical Systems, Best, the Netherlands) and/or with single slice dynamic SS-TSE or SSFP-sequences. The total examination time was about 45 min. Fetal MRI scans were reviewed at the time of acquisition by a radiologist, most often one of the authors (JW). The radiologist was informed about the ultrasound findings before the MRI examination. All MRI examinations were regarded to be of diagnostic quality. The obstetricians responsible for the clinical management were informed about the MRI findings.

Data concerning the participants such as age, BMI, number of pregnancies and deliveries, gestational age at ultrasound and at MRI, and results of fetal invasive procedures if performed were extracted from the medical records. For nine patients, the number of previous pregnancies could not be retrieved and for three the number of deliveries.

Antenatal ultrasound and MRI findings were reviewed in relation to the final diagnosis, which was based on the assessment of all available data, including postpartum clinical follow-up until discharge from the hospital, and autopsy results. If more than one ultrasound had been performed before the MRI, the last US was used for comparison.

The classification regarding added information was made in consensus between three of the authors (FC, OA, and JW) at a physical meeting and was classified into three categories:

I: MRI provided no additional information.
II: MRI provided additional information, either by detection of abnormalities not detected by ultrasound, or by provision of more comprehensive characterization of the detected anomaly, but did not change the management.
III: MRI provided additional information (see above), which changed the management.

### Statistical analysis

To evaluate the association between category and age, BMI, and oligohydramnios, we fitted an ordinal-logistic regression model with age, BMI, and oligohydramnios as explanatory variables, and an interaction term between BMI and oligohydramnios. *p*-Values were computed using the bootstrap with case-wise resampling. We also merged categories II and III and fitted a logistic regression model using the same explanatory variables. The analysis was performed using R 4.3.1^11^ and version 0.5 of the boot.pval package.^12^

#### Ethical approval

The study was approved by the National Swedish Ethics Committee.

## Results

### Characteristics of the patients

The mean age of the participants was 29 years (range 16–45). The mean number of pregnancies was two (range 1–5) and of deliveries 0.8 (range 0–5). The mean gestational age at the last US before MRI was 19 + 4 weeks (range 14 + 0–27 + 5). The mean BMI was 26.0 kg/m^2^ (range 19–46). In category I, eleven (14%) of the patients had a BMI >30 kg/m^2^, in category II four (15 %), and in category III four (25%). Oligohydramnios was seen in twelve (15%) of the cases in category I, three (12%) in category II, and six (38%) in category III. The median time between the last ultrasound and MRI was 3 days (range 0–35 days). In 83% of the cases, MRI was performed within 7 days of the US (Table 1).

**Table 1.** Distribution of cases with CNS- and non-CNS anomalies and the proportion of high BMI and oligohydramnios into different categories.

	Category			
	I	II	III	Total
CNS anomaly	35 (69%)	11 (22%)	5 (10%)	51
Non-CNS anomaly	44 (63%)	15 (21%)	11 (16%)	70
BMI > 30 Kg/m^2^	11 (14%)	4 (15%)	4 (25%)	19
Oligohydramnios	12 (15%)	3 (12%)	6 (38%)	21

### Distribution of cases in categories I–III

**Fig 1.**
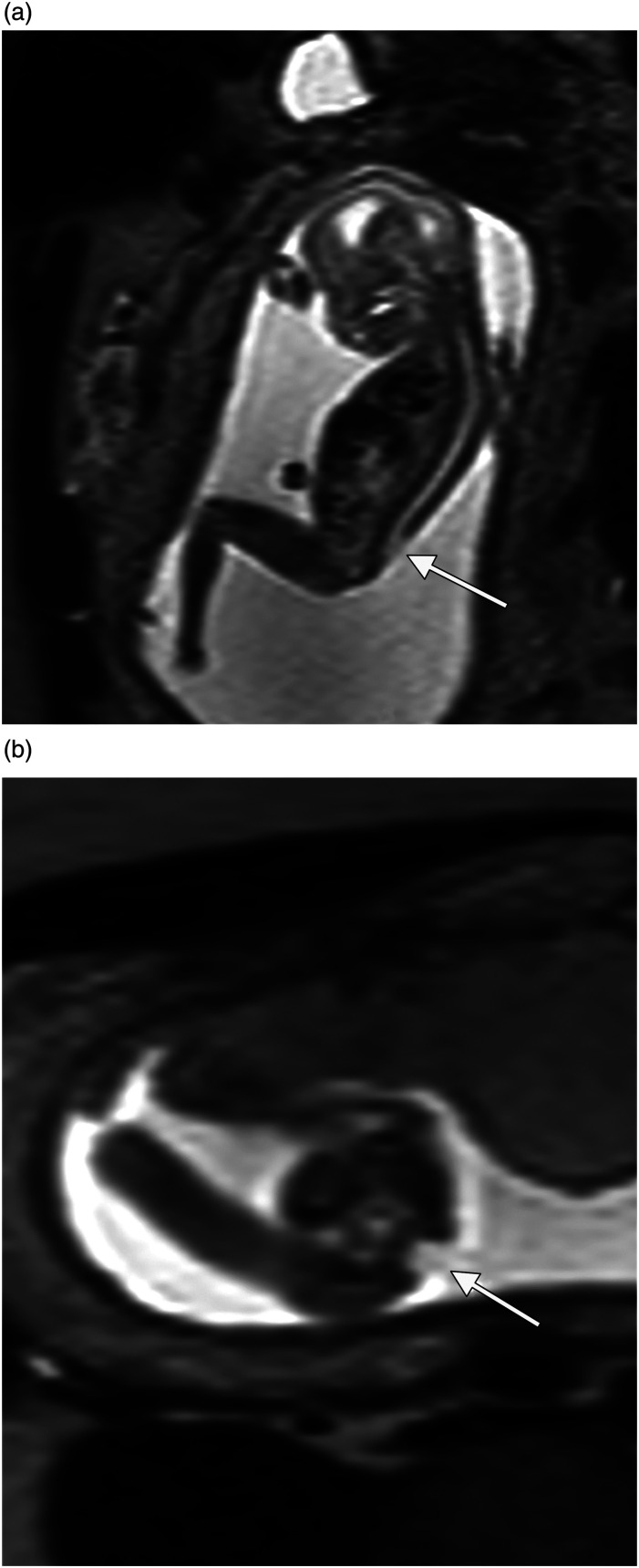
(a) and (b). Case no 2. Ultrasound examination revealed lemon-shaped head. Sagittal (a) and axial (b) MR images show low lying cerebellum but also lumbosacral spinal closure defect (arrows) as in myelomeningocele.

**Fig 2.**
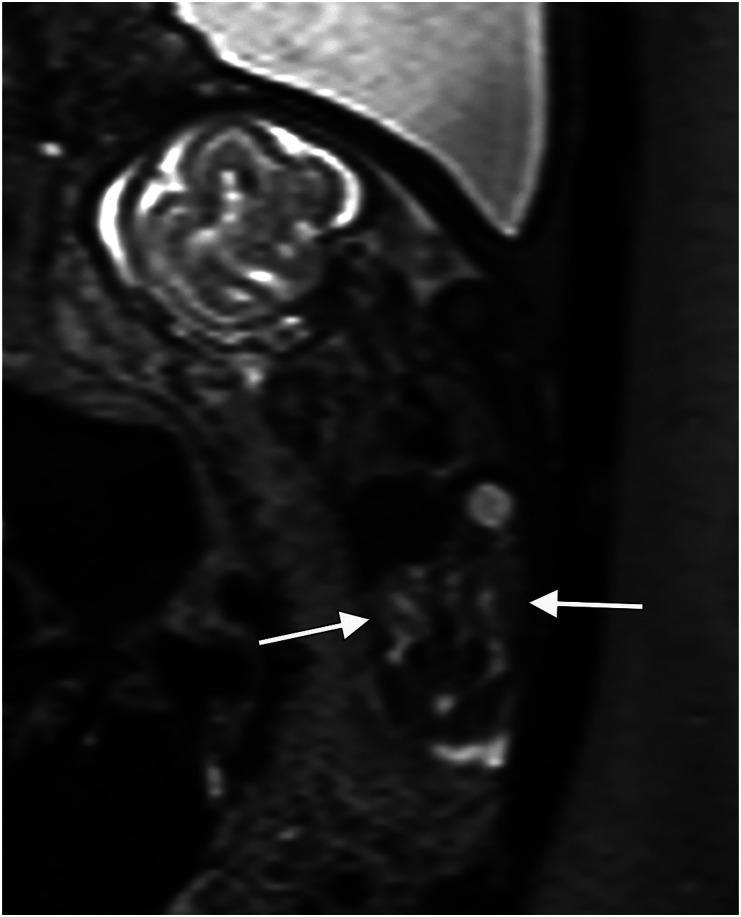
Case no 31. At ultrasound examination, there was a suspicion of polycystic kidney disease. Coronal MR image shows kidneys of normal size and signal intensity (arrows).

**Fig 3.**
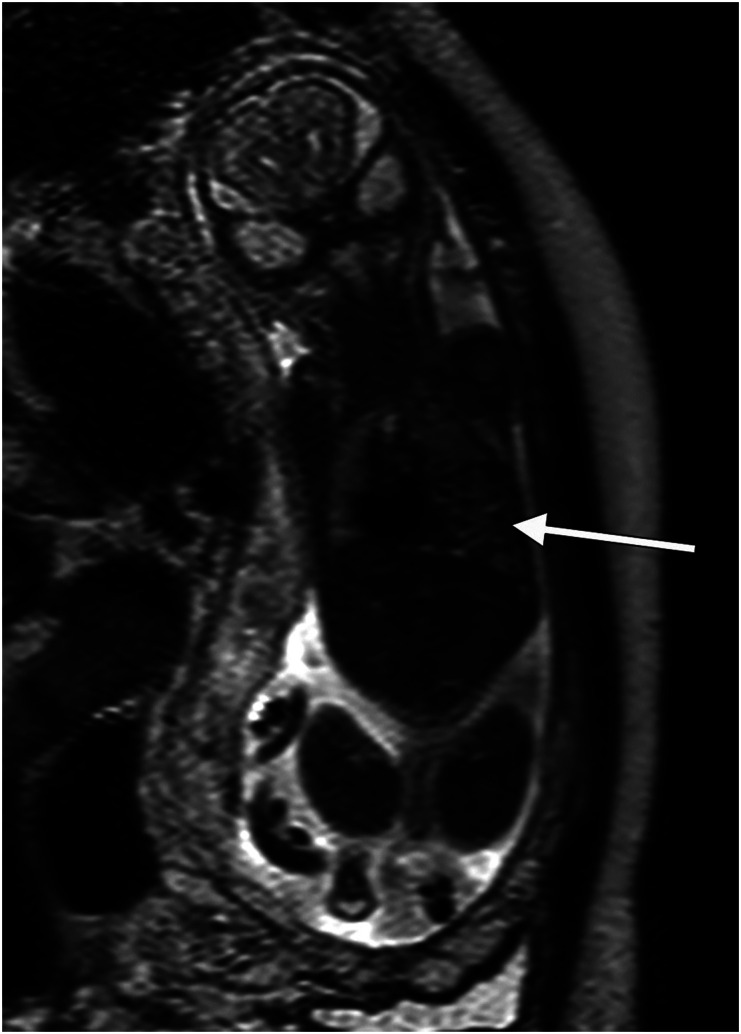
Case no 153. Ultrasound examination revealed right-sided heart but intact diaphragm. Coronal MR image shows left-sided diaphragmatic hernia containing intestines (arrow) but with normal position of the liver.

**Fig 4.**
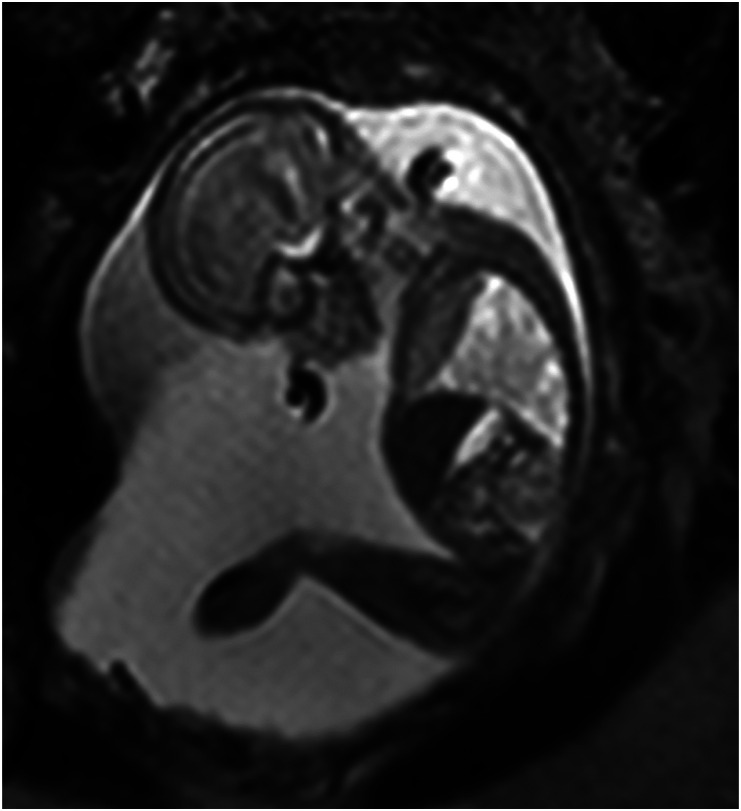
Case no 174. At ultrasound examination, there was a suspicion of left-sided diaphragmatic hernia. Sagittal MR image shows intact diaphragm, but signal intensity changes suggesting congenital cystic adenomatoid malformation in the inferior left lung lobe.

Distribution of cases is presented in Table 1. Of all CNS cases, MRI provided additional information without changing the management in eleven (22%) and revealed additional information which changed the management in five (10 %). The corresponding figures for non-CNS cases were 21 and 16%, respectively. Detailed information on the fetuses in category III is shown in Table 2. In five cases, the ultrasound findings were found to be false positive. All these pregnancies ended with liveborn healthy infants. Examples of MR images from category III are displayed in Figs. 1–4 (Fig. 1 here, two pictures, case no 2).

**Table 2.** Ultrasound (US) and magnetic resonance imaging (MRI) findings, the final outcome, and diagnosis for the 16 cases in category III.

Case number	BMI at booking (kg/m^2^)	US finding	MRI findings	Gestational age at US	Days between US and MRI	Final outcome	Diagnosis
2	28	Suspicion of “lemon sign.” Slight dilatation of the lateral ventricles	Myelomeningocele with herniation of cerebellum into foramen magnum. No hydrocephalus	17 + 3	1	Termination of pregnancy	Fetal autopsy: Lumbosacral myelomeningocele. Normal karyotype
3	26	Anhydramnios. Discrepancy between abdominal and head sizes. Increased nuchal thickness. Kidneys, bladder, and stomach not distinguishable. Possibly enlarged lungs	Renal agenesis and absent urinary bladder. Expanded lungs containing small cysts	18 + 3	8	Termination of pregnancy	Fetal autopsy: Renal agenesis. Congenital cystic adenomatoid malformation (CCAM). Foot deformities, vertebral defects in the lumbar region, and esophageal- and anal atresia
6	27	Oligohydramnios. Slightly divergent skull form. No signs of dilated ventricles. Normal configuration of the cerebellum. Spine intact. Heart, diaphragm, and abdominal wall without remarks. Stomach and bladder identified	Oligohydramnios. No normal kidneys distinguishable. Several cyst-like structures in the lower part of the abdomen. No distinguishable bladder. Left-sided cystic kidney?	15 + 6	1	Termination of pregnancy	Fetal autopsy: The left kidney dysplastic and partly cystic. Right-sided renal agenesis. Membranous covered anus. Small chin
24^a^	20	Dilation of the posterior horns of the lateral ventricles	Normal findings. The width of the lateral ventricles is within normal range	18 + 5	1	Liveborn infant	Healthy child
45	35	Oligohydramnios. Kidneys cannot be seen in detail. Flattened skull. Brain structures not possible to see in detail. Funnel-shaped thorax	Oligohydramnios. Kidneys and bladder cannot be identified. Normal CNS, lungs, heart, thorax, and liver	19 + 2	3	Termination of pregnancy	Fetal autopsy: Bilateral renal agenesis
31^a^	33	Discrete hydronephrosis and increased echogenicity that could indicate polycystic kidneys. Fluid-filled urinary bladder	No signs of polycystic kidneys. No other pathological changes	19 + 4	1	Liveborn infant	Healthy child
48	22	Oligohydramnios. One kidney identified. Above the fetal head, a cavity containing several septae	Oligohydramnios. Kidneys and bladder of normal shape and size	18 + 5	0	Liveborn infant	Emergency caesarean section in week 24 + 6. Placental abruption
							The child died due to lung hypoplasia. Preterm premature rupture of membranes?
69	21	Anhydramnios. Suspected urinary tract anomaly. The kidneys properly located. The bladder and stomach not identified	Anhydramnios. No kidneys identified. No fluid at the location of the bladder	16 + 5	4	Termination of pregnancy	Fetal autopsy: Renal agenesis and congenital high airway obstruction syndrome and other features corresponding to Frasers syndrome
93	Missing	Slight dilation of the lateral ventricles and third ventricle. No other deviant findings	Moderate dilation of the lateral and third ventricle. Undefined changes like calcification or blood clots near the lateral ventricles. Suspicion of infection or intracerebral hemorrhage	19 + 6	0	Termination of pregnancy	Fetal autopsy
							Dilated ventricles. Defective development of the cerebral gyri (status verrucosus) hemorrhages in the germinal matrix
106^a^	28	Oligohydramnios. Oblong head. CNS and extremities cannot be evaluated	Normal CNS findings	20 + 1	2	Liveborn infant	Healthy child
131^a^	19	Suspected defect of the vermis	Prominent cisterna magna. Cerebellum normal. No dilation of the ventricles. Normal spinal canal	19 + 3	7	Liveborn infant	Healthy child
148	34	Suspected arthrogryposis. Crossed lower extremities without movements	Caudal regression syndrome. Agenesis of sacrum	17 + 4	6	Termination of pregnancy	Fetal autopsy: Caudal regression syndrome. Deformed vertebral column and undeveloped pelvic bones and lower extremities
153	23	Right-sided displacement of the heart. The diaphragm and the abdominal wall normal	Diaphragmatic hernia, including intestines. Liver and stomach in proper positions. Right-sided displacement of the heart	18 + 0	15	Liveborn infant	Left-sided diaphragmatic hernia
167	Missing	Suspected left-sided diaphragmatic hernia including the stomach	Diaphragmatic hernia including intestines, stomach, and part of the liver. Right-sided displacement of the heart	17 + 6	6	Termination of pregnancy	Autopsy not performed
171	22	Banana sign. Lateral ventricles not possible to measure. No abnormal findings of the vertebral canal. Skin surface seems to be intact	Suspected lumbosacral myelomeningocele. Herniation of the cerebellum through foramen magnum	19 + 6	5	Liveborn infant	Lumbosacral myelomeningocele
174^a^	32	Suspected diaphragmatic hernia. Right-sided displacement of the heart and abdominal aorta. No signs of intestines, stomach, or cysts in the thorax	No signs of diaphragmatic hernia. A lesion with increased echogenicity in the left lung. Suspected congenital cystic adenomatoid malformation	18 + 6	6	Liveborn infant	Healthy child

^a^Cases with false-positive US findings.

### Interventional procedures

Invasive procedures (amniocentesis or chorionic villus sampling) were performed in 86 (72%) pregnancies. In category I, one fetus was diagnosed with trisomy 21, one with trisomy 13, and one with Fragile X-syndrome. In category II, one fetus was diagnosed with trisomy 21. In category III, 10 (63 %) invasive procedures were performed without adverse results (Fig. 2 here, case no 31).

### Pregnancy outcomes

Twenty-eight pregnancies (36%) in category I were terminated: one ended as intrauterine fetal death and forty-nine (63%) with liveborn infants. In category II, nine pregnancies (34%) were terminated, two (8%) ended as intrauterine fetal death, one as a missed abortion (4%), and fourteen (54%) with liveborn infants. In category III, eight pregnancies (50%) were terminated and eight (50%) ended with liveborn infants. (Figs. 3 and 4 here, case nos. 153 and 174).

In total, 49 (40%) pregnancies, with the same number of fetuses, led to termination of pregnancy, missed abortion, or intrauterine fetal death, where 34 (69%) of these fetuses were autopsied. In category III, seven (88%) out of the eight fetuses terminated were autopsied.

### Relation between gestational age, BMI, and oligohydramnios and categories

In ordinal logistic regression model, there were trends but no significant associations between the covariates (gestational age, BMI, and oligohydramnios) and the categories (Table 3).

**Table 3.** Ordinal-logistic model with categories I–III.

Variable	OR	*p*-Value
Gestational age at MR (days)	0.979	0.092
BMI > 30	2.722	0.085
Oligohydramnios	2.692	0.093
BMI > 30 and oligohydramnios	0.431	0.491

## Discussion

In this study, MRI provided additional information in 22% of all cases (CNS and non-CNS) without changing the management and revealed information that changed the management of the pregnancy in 13%. When a CNS anomaly was detected or suspected, MRI provided additional information in 22% of the cases without changing the management and revealed additional information which changed the management in 10 %. The corresponding numbers for non-CNS cases were 21% and 16 %, respectively. The proportion of cases where MRI provided added information that changed therapeutic decisions was larger in the groups with maternal obesity (25%) and oligohydramnios (38%), although not significantly so.

Our finding is of special importance in countries where there is a gestational age limit for pregnancy termination.

Previous studies have suggested that MRI mainly has a complementary role in detecting CNS anomalies.^3,5^ Rossi et al. conducted a review, including 13 articles and 710 fetuses.^13^ MRI provided additional information which changed the management of the pregnancy in 30%. Gonçalves et al.^3^ reported a similar figure of 22%. Our figure, 10%, is substantially lower but compares well with 11% reported by Rodríguez et al.^5^ and 10% from our previous report.^9^ It should, however, be pointed out that change of management can partly be seen as a question of subjective assessment. Moreover, we only included fetuses from the second trimester, in contrast to the other referred publications. Verburg et al. included both CNS and non-CNS anomalies and reported that MRI changed the management in 8%,^14^ a figure in the same range as our 13%. Also, Rodríguez et al. showed that MRI has an important role in providing useful information for non-CNS anomalies such as fetal abdominal- and thoracic malformations.^5^ Our figure concerning change of management for non-CNS anomalies, 16%, compares well with their findings. When comparing results from different studies regarding proportions with added information, it should also be acknowledged that these numbers will vary with local referral routines. More selective use of MRI will probably increase the number with added information.

In our previously published studies, including all patients with a suspected or detected fetal anomaly at the routine ultrasound in the second trimester,^9,10^ we were able to show that MRI seemed to contribute with clinically valuable information, especially in cases with maternal obesity and oligohydramnios. These findings are now supported by the results in our present study with substantially larger numbers, with numerically higher proportions of cases with added value from MRI in cases of maternal obesity and oligohydramnios. Moreover, we saw a trend for an effect of gestational age on the presence of an added value.

Today, ultrasound is the standard screening method to detect fetal anomalies. It is cost-effective, easily available, and shows real-time images. Fadda et al. showed that the overall sensitivity to detect fetal anomalies at the routine ultrasound screening in the second trimester was 55% and the specificity 99%.^15^ Most probably, the sensitivity is somewhat higher nowadays.

False-positive ultrasound diagnoses of fetal anomalies are a reality^16^ and a worst case scenario is a termination of a pregnancy with a fetus without or with a less severe anomaly than the patient had been informed about. We had no cases of false-positive MR findings but such results have been reported in a small proportion of cases in the literature.^13^

In Table 2, five cases are presented where MRI could rule out anomalies suspected at ultrasound. All five cases ended up as healthy children. The suspected anomalies included diaphragmatic hernia, polycystic kidneys, and three cases of suspected CNS anomalies. Thus, MRI can help clinicians to avoid terminations of pregnancies due to incorrect diagnoses and thus provide valuable information to the patient. Moreover, as also reflected in Table 2, MRI can improve diagnostics of fetal anomalies, thereby optimizing the information given to pregnant women as well as the management of the pregnancies.

The additional information received in the category II cases is also valuable, as it can contribute to more reliable fetal diagnoses and thus improve the counseling concerning the couple’s forthcoming pregnancies.

We were able to include 120 patients and 121 fetuses, which is a high number for this kind of study. There is a reason to believe that the time between the US and MRI is crucial for a fair comparison between the ultrasound and MRI findings. In our study, the median time between the ultrasound and MRI was 3 days, and 83% performed MRI within 1 week of the ultrasound. Some studies have reported on longer time intervals.^7,17^ Our results are well in line with Kul et al.^4^ who performed 95% of the MRI examinations within 3 days and Whitby et al.^18^ with 100% performed within 4 days.

The study included only second trimester fetuses, which we see as a strength. As pointed out by Millischer et al.,^19^ MRI in the second trimester involves special challenges including increased fetal movements. Early detection of fetal anomalies is imperative, especially in countries with a gestational age limit for termination of a pregnancy. Moreover, early detection of an anomaly is a prerequisite to correctly monitor the pregnancy and plan for optimal delivery in case the patient decides to fulfill the pregnancy. Another strength is that a large number of the terminated pregnancies was autopsied, 88% in category III.

There are some limitations of the present study that should be acknowledged. A limitation is the retrospective study design, meaning that data were collected from medical records. Due to findings in our previous publications,^9,10^ only selected cases were sent for MRI. This can be seen as a selection bias but, on the other hand, it is a reflection of how new knowledge is implemented in clinical practice. About 10 years have elapsed since the study examinations were performed and the quality of the ultrasound or MRI examinations has changed marginally and for both modalities. The same protocols are used today. We did not perform neurosonography by vaginal ultrasound, which can provide additional information to abdominal ultrasound.^17^ Thus, neurosonography might have implied an improved performance of ultrasound. However, we performed ultrasound examinations as it is usually done in Sweden. Moreover, the study material consists of a heterogeneous group of diagnoses, and the study size does not permit sub-group analyses.

In conclusion, our previous findings could be validated. MRI complements ultrasound and improves diagnosis of fetal CNS- and non-CNS anomalies especially when oligohydramnios or maternal obesity is present.
